# Identification and verification of prognostic cancer subtype based on multi-omics analysis for kidney renal papillary cell carcinoma

**DOI:** 10.3389/fonc.2023.1169395

**Published:** 2023-04-05

**Authors:** Baodong Wang, Mei Li, Rongshan Li

**Affiliations:** ^1^ Department of Nephrology, Fifth Hospital of Shanxi Medical University (Shanxi Provincial People’s Hospital), Taiyuan, China; ^2^ Department of Laboratory Medicine, Shanxi Provincial Hospital of Integrated Traditional Chinese and Western Medicine, Taiyuan, China

**Keywords:** kidney renal papillary cell carcinoma, prognosis, immune microenvironment, drug response, multi-omics

## Abstract

**Background:**

Identifying Kidney Renal Papillary Cell Carcinoma (KIRP) patients with high-risk, guiding individualized diagnosis and treatment of patients, and identifying effective prognostic targets are urgent problems to be solved in current research on KIRP.

**Methods:**

In this study, data of multi omics for patients with KIRP were collected from TCGA database, including mRNAs, lncRNAs, miRNAs, data of methylation, and data of gene mutations. Data of multi-omics related to prognosis of patients with KIRP were selected for each omics level. Further, multi omics data related to prognosis were integrated into cluster analysis based on ten clustering algorithms using MOVICS package. The multi omics-based cancer subtype (MOCS) were compared on biological characteristics, immune microenvironmental cell abundance, immune checkpoint, genomic mutation, drug sensitivity using R packages, including GSVA, clusterProfiler, TIMER, CIBERSORT, CIBERSORT-ABS, quanTIseq, MCPcounter, xCell, EPIC, GISTIC, and pRRophetic algorithms.

**Results:**

The top ten OS-related factors for KIRP patients were annotated. Patients with KIRP were divided into MOCS1, MOCS2, and MOCS3. Patients in the MOCS3 subtype were observed with shorter overall survival time than patients in the MOCS1 and MOCS2 subtypes. MOCS1 was negatively correlated with immune-related pathways, and we found global dysfunction of cancer-related pathways among the three MOCS subtypes. We evaluated the activity profiles of regulons among the three MOCSs. Most of the metabolism-related pathways were activated in MOCS2. Several immune microenvironmental cells were highly infiltrated in specific MOCS subtype. MOCS3 showed a significantly lower tumor mutation burden. The CNV occurrence frequency was higher in MOCS1. As for treatment, we found that these MOCSs were sensitive to different drugs and treatments. We also analyzed single-cell data for KIRP.

**Conclusion:**

Based on a variety of algorithms, this study determined the risk classifier based on multi-omics data, which could guide the risk stratification and medication selection of patients with KIRP.

## 1 Introduction

Renal cell carcinoma (RCC) is a common malignancy of urinary system (1). Clear cell carcinoma (ccRCC) is the most common pathological type of RCC, accounting for about 70% of RCC, followed by Kidney Renal Papillary Cell Carcinoma (KIRP) and chromophobe renal cell carcinoma (1). Although surgical resection is a good treatment for renal cell carcinoma at early stage, 30% of patients are diagnosed in advanced stage due to delayed diagnosis, and 10% to 20% of patients develop metastatic kidney cancer due to postoperative recurrence, which eventually leads to death (2–4). At present, due to the lack of understanding of the pathogenesis of renal cancer, there is a lack of effective treatment for metastatic renal cancer. Therefore, to explore the pathogenesis of kidney cancer is of great significance for the treatment of kidney cancer. The search for biomarkers related to kidney cancer can help clinicians personalize patient treatment strategies and increase patient benefits.

Kidney cancer is a heterogeneous disease with multiple subtypes, multiple genes, different biochemical characteristics and multiple forms (5). KIRP, the second most common type of RCC, is a heterogeneous disease originating in the tubular epithelium of the kidney (6). The histological features of KIRP are the papillary arrangement of tumor cells, and the axis of the papilla is fibrous vascular tissue (7). In 1997, Delahunt and Eble divided KIRP into type 1 and type 2 according to histopathological characteristics and prognostic differences (8). In many studies, histological subtypes have been shown to be important prognostic predictors, with type 1 KIRP having a better prognosis than type 2 KIRP (9). Previous studies have reported that type 2 KIRP have higher nuclear grading, later staging, and poorer prognosis than type 1 KIRP (10). In contrast, Bigot et al. showed in a study of 486 patients with KIRP who underwent nephron-sparing surgery that the histological subtype of KIRP had no effect on postoperative tumor outcome (11). In conclusion, whether the histological subtype involved in type 1 or type 2 can be used as an independent prognostic factor is controversial, and correct histological phenotype and prognostic prediction are essential for the formulation of medical protocols.

Advances in sequencing technology and machine learning of all kinds have led to significant advances in the acquisition and analysis of omics data, which have deepened the understanding of tumors at the molecular level (12). Compared with a single type of data, omics data reflect the characteristics of biological individuals at multiple levels, which provides the possibility to delineate cellular molecular mechanisms in detail. Different levels of omics data reflect different relationships between genomic distribution, cancer occurrence, progression, and prognosis (13). At the same time, each omics data has its own advantages. For example, methylation chip data and lncRNA expression matrix have good tissue conservation, which can be used as efficient markers for the early diagnosis of specific tumor tissues (14). miRNA data are characterized by dissociation and can be used for non-invasive diagnosis and dynamic detection of disease (15). Common transcriptome, or mRNA sequencing, is the cheapest and most readily available, and is suitable for use in a wide range of cohort studies to explore general patterns in patient populations (16–25).

RCCs with different pathological types have different therapeutic methods and prognosis. In addition, existing targeted drugs are mainly used for ccRCCs, with unclear clinical efficacy in non- ccRCCs (26, 27). It is important to note that there is currently a lack of multi-omics prognostic molecular typing based on KIRP to guide the diagnosis and treatment of KIRP. In this study, the risk stratification of KIRP was studied by integrating multiple omics, and the differences of subgroups were analyzed in each single omics data to characterize the key events in the development of KIRP. The study provides a reference for precision medicine of KIRP.

## 2 Materials and methods

### 2.1 Extraction and preprocessing of multi-omics data for KIRP

The dataset for KIRP was downloaded from The Cancer Genome Atlas (TCGA) (28) and TCGA database had the multi-omics data for our analysis in this study. We acquired gene expression profile for transcriptomics (including mRNAs encoding protein, long noncoding RNAs as known as lncRNAs, microRNA known as miRNAs, data of methylation, and data of gene mutations). We applied TCGAbiolinks package of R application to acquire clinicopathologic information and multi omics-based data. We downloaded the gene expression profiles of 34 cases with KIRP from GSE2748 as the external validation cohort (29). The patients with KIRP in the GSE2748 dataset had the prognostic information (29). In addition, we searched and downloaded the single-cell RNA sequencing for KIRP from GSE152938 (30). There was a total of four KIRP samples and one normal kidney sample included in GSE152938 (30). The matrix for single-cell RNA sequencing was generated by R package Seurat (31).

### 2.2 Identification of multi omics-based cancer subtypes by integrative analysis

The MOVICS package aimed to show the multi-omics comprehensive clustering and visualization of cancer typing studies (32). There were ten algorithms included in the MOVICS package: CIMLR, iClusterBayes, MoCluster, COCA, ConsensusClustering, IntNMF, LRAcluster, NEMO, PINSPlus and SNF (32). For the multi omics-based data, we focused on the characteristic related to prognosis (OS). The OS-related features, including mRNAs, lncRNAs, miRNAs, methylation, and gene mutations were analyzed by Univariate Cox regression analysis, and we screened out features with the threshold P-value<0.05. Due to the small amount of mutation matrix and miRNA expression data, only the top 30 mutations and 200 miRNA data were extracted. We carried out analysis for Clustering Prediction Index (CPI) (33) and Gaps-statistics (33) to filtrate out the optimal number of cancer subtypes. We finally identified the multi omics-based cancer subtype (MOCS) based on consensus ensembles and high robustness, thus separating the patients with KIRP into different MOCSs.

### 2.3 Nearest template prediction validation

Nearest template prediction (NTP) algorithm could also be applied to cross-platform, cross-species and multi-class predictions without any optimization of analysis parameters (34). In this study, we also used NTP algorithm of CMScaller package to test the dependability and stability of MOCS subtypes *via* the external GSE2748 cohort.

### 2.4 Biological characteristics for MOCS subtypes

The gene sets (including immune-related pathways) were analyzed, and enrichment scores were calculated using gene set variation analysis (GSVA) from R package GSVA (35). The differentially expressed genes (DEGs) among the three MOCS subtypes were assessed using limma package (36). Pathway enrichment analysis was performed by clusterProfiler package with the employment of Biological Processes in Gene Ontology (GO) (37).

### 2.5 Calculation of immune microenvironmental cell abundance and immune checkpoint

Tumor Immune Estimation Resource (TIMER) is a website from which researchers can use RNA-Seq expression profile data to detect the infiltration of immune cells in tumor tissue (38). The TIMER provides the infiltrations of six kinds of immune cells (B cells, CD4^+^ T cells, CD8^+^ T cells, Neutrophil, Macrophages and Myeloid dendritic cells) (38). CIBERSORT (39) and CIBERSORT-ABS (40) algorithms were used to acquire the infiltrations of 22 kinds of immune cells. quanTIseq is a deconvolution tool developed specifically for RNA-seq data, enabling accurate quantification of unknown tumor content, as well as the immune cell component of the overall tissue (41). quanTIseq implemented a complete deconvolution process for analyzing RNA-seq data based on constrained least squares regression and a new eigenmatrix from 51 purified or enriched RNA-seq data sets, avoiding inconsistencies between mixtures and eigenmatrices (41). MCPcounter (42), xCell (43), and EPIC (44) algorithms (Estimate the Proportion of Immune and Cancer cells) were also used to assess the immune microenvironmental cell abundance. We estimated the infiltrating level of immune or stromal scores using ESTIMATE R package (45). In addition, DNA methylation of tumor-infiltrating lymphocyte (MeTIL) for TCGA- KIRP cohort was also calculated (46).

### 2.6 Evaluation of genomic mutation for MOCS subtypes

Mutation profiles of KIRP were acquired and we compared and visualized the difference of mutation among the MOCS subtypes utilizing Maftools package of R (47). We applied the Maftools function to analyze the oncogenic pathway and mutually exclusive or coexisting mutations (48). The loss and gain in genomic level was evaluated by GISTIC 2.0 algorithm (49).

### 2.7 Drug sensitivity profiles for MOCS subtypes

R package pRRophetic was employed to predict the drug sensitivity profiles for MOCS subtypes (50, 51). Subclass mapping was used to explore the immunotherapy of KIRP based on the literature published (52, 53).

### 2.8 Statistical analyses

R was used to conduct statistical analyses (v4.0.2). We also provide the codes of all methods used in this paper in Supplementary Code. P values or adjusted P values less than 0.05 were considered significant for all statistical comparisons.

## 3 Results

### 3.1 Three MOCSs were categorized for KIRP patients by MOVICS package

**Figure 1:**
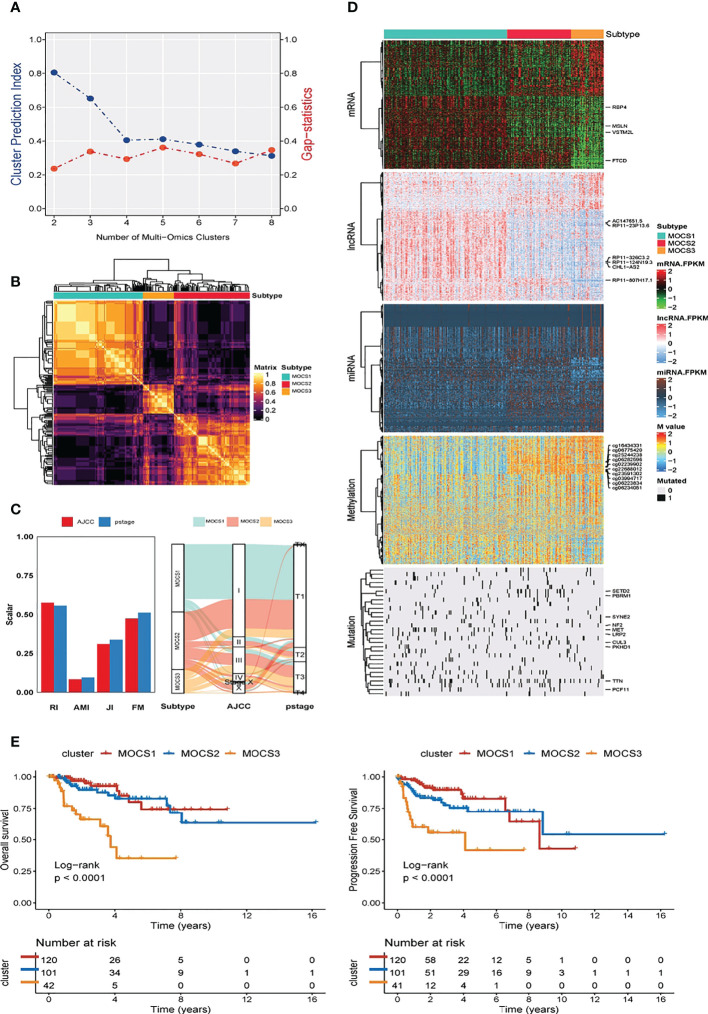
Three MOCSs were categorized for KIRP patients by MOVICS package. **(A)** Determination of optimal cluster number through calculating CPI (blue line) and Gaps-statistics (red line) in TCGA- KIRP cohort. **(B)** Consensus heatmap based on outcomes from 10 multi-omics integrative clustering approaches with subtype number of three showing perfect diagonal rectangle. **(C)** Quantification of sample similarity using silhouette score based on the consensus ensembles result and alluvial diagram presenting the flow distribution among different multi omics-based cancer subtypes (MOCSs). **(D)** Comprehensive heatmap showing the detailed molecular landscape multi-omics data for mRNA, lncRNA, miRNA, DNA methylation, and gene mutations among the three MOCSs. **(E)** log-rank test for overall survival time and progression free survival time for patients with KIRP.

We discovered three MOCS subtypes for KIRP patients based on CPI analysis and Gaps-statistics, due to the optimal average statistic value with the number of MOCSs was found to be three **(** **Figure 1A** **)**. Hence, patients with KIRP were divided into MOCS1, MOCS2, and MOCS3, indicating the robustness of the classification system **(** **Figure 1B** **)**. The silhouette plot indicated that the silhouette score of MOCS1 was 0.70, the silhouette score of MOCS2 was 0.42, while the silhouette score of MOCS3 was 0.71, which substantiated that the MOCS subtypes were distinguishable and separated well from each other **(** **Supplementary Figure S1A** **)**. From the **Figure 1C**, the consistency of the classification system for MOCSs were observed in consideration of four statistics (54) **(** **Figure 1C** **)**, including Rand Index (RI), Adjusted Mutual Information (AMI), Jaccard Index (JI), and Fowlkes-Mallows (FM) (54). Furthermore, we observed that all the patients in the MOCS1 and MOCS1 were at AJCC Stage I and Pstage I **(** **Figure 1C** **)**. Among the three MOCSs, we displayed the distribution of the multi-omics data for mRNA, lncRNA, miRNA, DNA methylation, and gene mutations as shown in the heatmap **(** **Figure 1D** **)**. In the distribution diagram **(** **Figure 1D** **)**, RBP4, MSLN, VSTM2L, FTCD, AC147651.5, RP11-23P13.6, RP11-326C3.2, RP11-124N19.3, CHL1-AS2, and RP11-807H17.1 were the top ten OS-related factors of transcriptome (mRNAs and lncRNAs). As for miRNA, hsa-mir-127, hsa-mir-1247, hsa-mir-1-1, hsa-mir-1-2, hsa-mir-1180, hsa-mir-1269a, hsa-mir-10b, hsa-mir-126, hsa-mir-105-1, and hsa-mir-105-2 were the top ten OS-related factors of miRNAs **(** **Figure 1D** **)**. As for DNA methylation, cg16434331, cg06775420, cg25244238, cg06282596, cg02239902, cg22688012, cg23591302, cg03994717, cg06223834, and cg06234051 were the top ten OS-related factors **(** **Figure 1D** **)**. SETD2, PBRM1, SYNE2, NF2, MET, LRP2, CUL3, PKHD1, TTN, and PCF11 were the top ten OS-related factors **(** **Figure 1D** **)**. Further, we compared the outcome of clinical prognosis of patients with KIRP among MOCS1, MOCS2, and MOCS3. Patients in the MOCS3 subtype were observed with shorter overall survival time than patients in the MOCS1 and MOCS2 subtypes **(** **Figure 1E** **)**, which was also observed for progression free survival time **(** **Figure 1E** **)**. Using NTP algorithm, three MOCSs were also identified as predicted by the external GSE2748 cohort **(** **Supplementary Figure S1B** **)**. Patients in the MOCS3 subtype were observed with shorter overall survival time **(** **Supplementary Figure S1C** **)**.

### 3.2 Biological characteristics for MOCS subtypes

**Figure 2:**
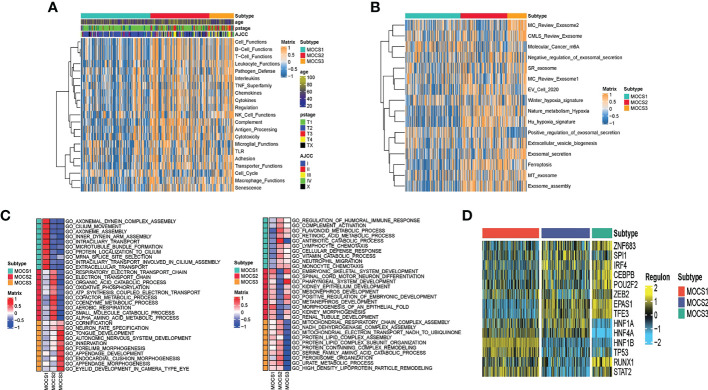
Biological characteristics for MOCS subtypes. **(A)** Heatmap showing GSVA enrichment score of immune-related pathways among the three MOCS subtypes. **(B)** Heatmap showing GSVA enrichment score of cancer-related pathways among the three MOCS subtypes. **(C)** GO enrichment analysis showing the upregulated pathways and the downregulated pathways. **(D)** Heatmap showing the regulon distribution among the three MOCS subtypes.

Further, we depicted the molecular features characterization for MOCS subtypes. We computed the enrichment score of immune-related pathways (including Cell Functions, B Cell Functions, T Cell Functions, Leukocyte Functions, Pathogen Defense, Interleukins, TNF Superfamily, Chemokines, Cytokines, Regulation NK Cell Functions Complement, Antigen Processing, Cytotoxicity, Microglial Functions, TLR, Adhesion, Transporter Functions, Cell Cycle, Macrophage Functions and Senescence) based on GSVA analysis. We could find that MOCS1was negatively correlated with immune-related pathways **(** **Figure 2A** **)**. As for other pathways, we found global dysfunction of cancer-related pathways among the three MOCS subtypes **(** **Figure 2B** **)**. Generally, MOCS1 showed relatively lower enrichment level of Nature metabolism Hypoxia, Hu hypoxia signature, Exosomal secretion, Ferroptosis, MT exosome and exosome assembly **(** **Figure 2B** **)**, suggesting the three MOCS subtypes were association with exosomes strongly. Biological processes of AXONEMAL DYNEIN COMPLEX ASSEMBLY, CILIUM MOVEMENT, AXONEME ASSEMBLY, INNER DYNEIN ARM ASSEMBLY, INTRACILIARY TRANSPORT, MICROTUBULE BUNDLE FORMATION, PROTEIN LOCALIZATION TO CILIUM, MRNA SPLICE SITE SELECTION, INTRACILIARY TRANSPORT INVOLVED IN CILIUM ASSEMBLY, and EXTRACELLULAR TRANSPORT were overactivated in MOCS1 **(** **Figure 2C** **)**. Biological processes of RESPIRATORY ELECTRON TRANSPORT CHAIN, RESPIRATORY ELECTRON TRANSPORT CHAIN, ELECTRON TRANSPORT CHAIN, ORGANIC ACID CATABOLIC PROCESS, OXIDATIVE PHOSPHORYLATTON, GOTATP SYNTHESIS COUPLED ELECTRON TRANSPORT, COFACTOR METABOLIC PROCESS, COENZYME METABOLIC PROCESS, AEROBIC RESPIRATION, SMALL MOLECULE CATABOLIC PROCESS, and ALPHA AMINO ACID METABOLIC PROCESS **(** **Figure 2C** **)**. Biological processes of CORNIFICATION, NEURON FATE SPECIFICATION, TONGUE DEVELOPMENT, AUTONOMIC NERVOUS SYSTEM DEVELOPMENT, INNERVATION, FORELIMB MORPHOGENESIS, APPENDAGE DEVELOPMENT, ENDOCARDIAL CUSHION MORPHOGENESIS, APPENDAGE MORPHOGENESIS, EYELID DEVELOPMENT IN CAMERA TYPE EYE **(** **Figure 2C** **)**. In addition, we evaluated the activity profiles of regulons among the three MOCSs, thus highlighting the additional potential regulatory differences. The higher level of several regulon, such as ZNF683, IRF4, CEBPB, EPAS1, and TFE3 was observed in MOCS2 and MOCS3 **(** **Figure 2D** **)**, indicating the important differentiators of epigenetically driven transcriptional networks among the three MOCS subtypes. GSVA analysis was carried out regarding metabolism-related pathways, we found that most of the metabolism-related pathways were activated in MOCS2 **(** **Supplementary Figure S2A** **)**. Consistently, most of immune-associated signatures were enriched in MOCS2 **(** **Supplementary Figure S2B** **)**.

### 3.3 Calculation of immune microenvironmental cell abundance and immune checkpoint

**Figure 3:**
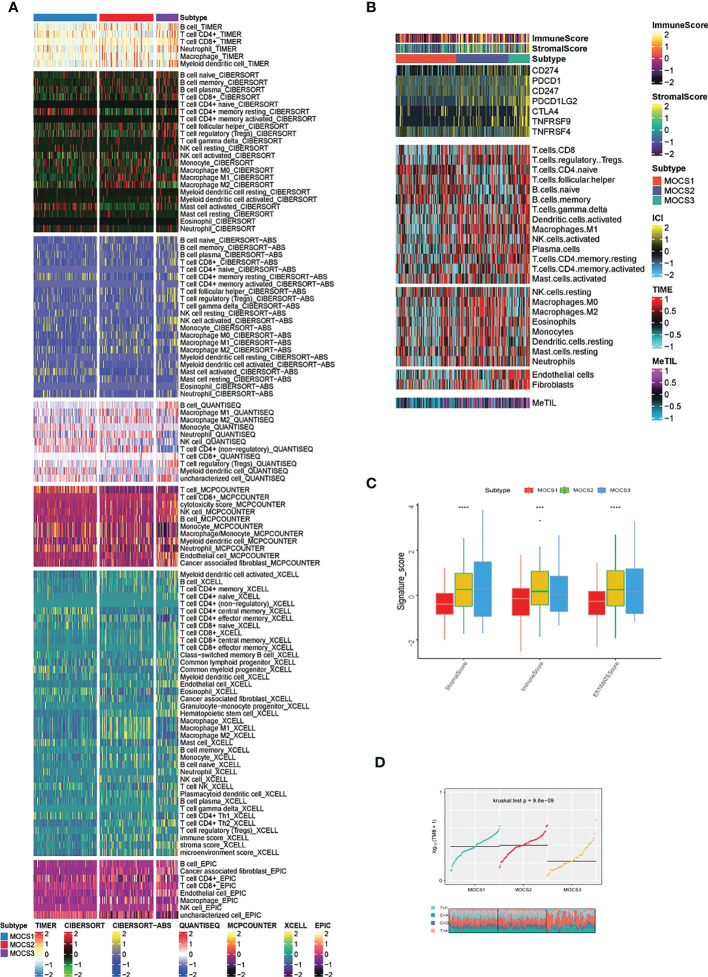
Calculation of immune microenvironmental cell abundance and immune checkpoint. **(A)** Heatmap showing the immune microenvironmental cell profile for TCGA- KIRP cohort based on TIMER, CIBERSORT, CIBERSORT-ABS, QUANTISEQ, MCPCOUNTER, XCELL, EPIC algorithms. **(B)** Heatmap showing the profile for immune checkpoint genes, and DNA methylation of tumor-infiltrating lymphocytes (MeTILs). **(C)** Boxplot showing the distribution of immune, stromal and ESTIMATE scores. **(D)** Distribution of TMB and TiTv (transition to transversion) among the three MOCS subtypes. *** means P < 0.001 and **** means P < 0.0001.

In consideration of the critical role of immunity in KIRP progression, we investigated the immune microenvironmental cell abundance and immune checkpoint among the three MOCS subtypes. Several immune microenvironmental cells were highly infiltrated in specific MOCS subtype. For instance, B cell in MOCS3, Macrophage M2 in MOCS2, NK cell in MOCS1 and so on **(** **Figure 3A** **)**. As for the immune checkpoint genes, on the whole, MOCS3 was associated with higher levels of immune checkpoint genes **(** **Figure 3B** **)**. MOCS3 was also associated with higher levels of MeTIL **(** **Figure 3B** **)**. MOCS1 was found to be associated with lower levels of immune, stromal and ESTIMATE scores **(** **Figure 3C** **)**. Additionally, we found that MOCS3 showed a significantly lower tumor mutation burden (TMB, **Figure 3D**). MOCS1 was found to be associated with lower signature score of CD8^+^ T effector, Immune checkpoint, APM, TME score A, Pan F TBRs, EMT2, EMT3, and TME score B **(** **Supplementary Figure S3A** **)**. The level of RNAss, DMPss, ENHss, EREG.EXPss and HRD was found to be lower in MOCS1 **(** **Supplementary Figure S3B** **)**.

### 3.4 Evaluation of genomic mutation for MOCS subtypes

**Figure 4:**
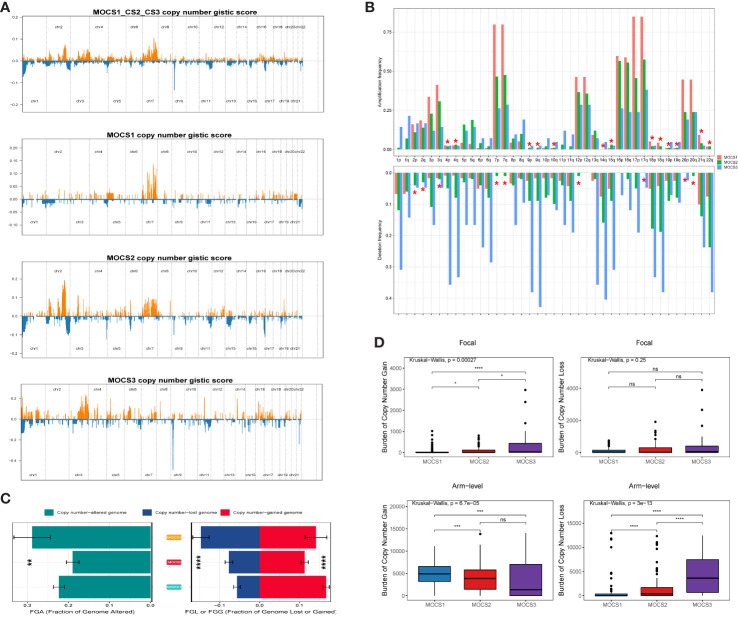
Landscapes of copy number variations. **(A)** Comparison of overall copy number among all patients with KIRP, MOCS1, MOCS2, and MOCS3. **(B)** The amplification or deletion frequency in chromosome among the three MOCSs. **(C)** Bar-plot indicating the total alteration frequency among the three MOCSs. **(D)** Different burden of copy number gain at focal and arm-level among the three MOCSs. * means P < 0.05, ** means P < 0.01, *** means P < 0.001, and **** means P < 0.0001, ns, no significance.

The differences in copy number variations (CNV) among the three MOCS subtypes were compared, and the result revealed that the CNV occurrence frequency was higher in MOCS1 **(** **Figures 4A–C** **)**. In detail, amplification in chr 2p, 2q, 3p, 3q, 4p, 7p, 7q, 12p, 12q, 16p, 16q, 17p, 17q, 18p, 18q, 20p, 20q, and 21q were higher in MOCS1 **(** **Figure 4B** **)**. The above results were also proved by the total copy number alteration rate as shown in **Figure 4C**. MOCS1 displayed a higher rate in focal and arm-level mutation level gain **(** **Figure 4D** **)**. Mutation patterns of the top 20 most frequently mutated genes among the three MOCSs were displayed in the waterfall plot **(** **Supplementary Figure S4A** **)**, from which we could see that, TTN, MET, CUBN, SYNE1, HERC2, KIAA1109, MUC16, PKHD1, WDFY3, DNAH8, KMT2C, LRP2, MACF1, NEB, PCLO, SMARCA4, ANK3, COL18A1, DDX5, DYNC2H1 were the top 20 mutated genes for MOCS1; TTN, SETD2, MUC16, CUL3, KIAA1109, KMT2C, PBRM1, PCF11, BAP1, FAT1, KMT2D, PKHD1, KDM6A, LRBA, SRRM2, ARID1A, ASAP2, BIRC6, CENPE and CNOT1 were the top 20 mutated genes for MOCS2; NF2, TTN, TXNIP, BAP1, CAMK1D, CDH8, CMYA5, CREBBP, EBF2, HECTD4, ITGAL, KRAS, MAP1B, TAS1R2, TG, EIF4G3, FAT1, HELZ2, KDM6A, and SYNE1 were the top 20 mutated genes for MOCS3 **(** **Supplementary Figure S4A** **)**. The synthetic lethal mutations in MOCS1, MOCS2, and MOCS3 were displayed in **Supplementary Figure S4B**. The potential druggable gene categories from the mutation dataset for MOCS1, MOCS2, and MOCS3 were shown in **Supplementary Figure S4C**, we found that ANK3, CUBN, LRP2, MET, PKHD1 and so on were the potential therapeutic targets for MOCS1; ARID1A, BAP1, CUL3, FAT1, KDM6A and so on were the potential therapeutic targets for MOCS2; BAP1, CAMK1D, CMYA5, CREBBP, FAT1 and so on were the potential therapeutic targets for MOCS3. The fraction of pathways and samples affected were the minimum among the three MOCSs **(** **Supplementary Figure S4C** **)**.

### 3.5 Drug sensitivity profiles for MOCS subtypes

**Figure 5:**
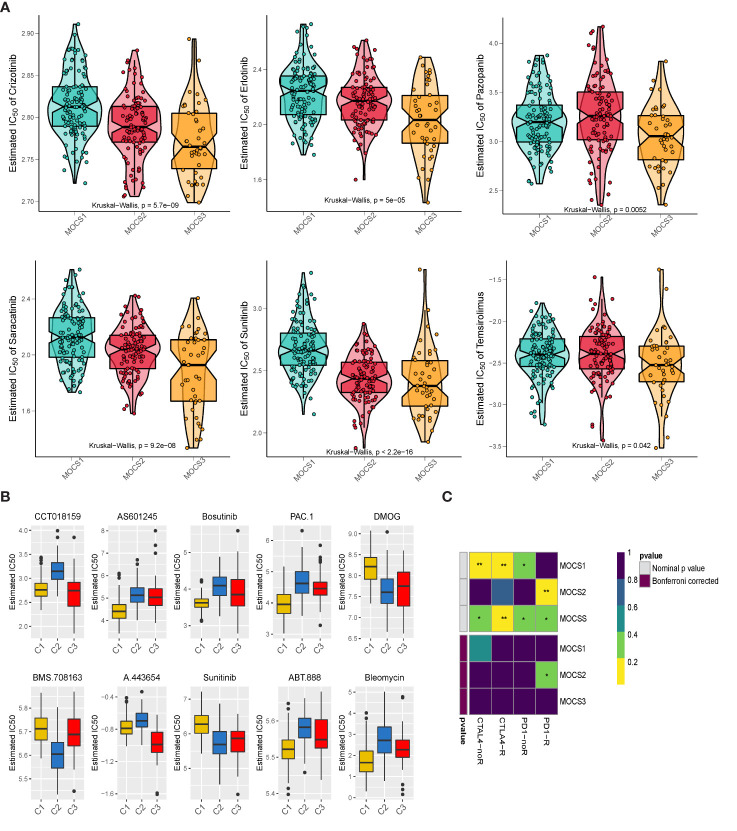
Drug Sensitivity Profiles for MOCS subtypes. **(A)** Estimated IC50 of Crizotinib, Erlotinib, Pazopanib, Saracatinib, Sunitinib, and Temsirolimus among the three MOCSs. **(B)** Estimated IC50 of CCT018159, AS601245, Bosutinib, PAC.1, DMOG, BMS.708163, A.443654, Sunitinib, ABT.888, and Bleomycin. **(C)** Subclass analysis manifested that MOCS2 were more likely to respond to anti-PD1 blockades. * means P < 0.05 and ** means P < 0.01.

We collected drug response data reflected by the IC50 value *via* GDSC database. We observed that patients in MOCS3 were more sensitive to Crizotinib, Erlotinib, Pazopanib, Saracatinib, Sunitinib, and Temsirolimus **(** **Figure 5A** **)**. We found that patients in MOCS1 were more sensitive to AS601245, Bosutinib, PAC.1, ABT.888, and Bleomycin **(** **Figure 5B** **)**. Whereafter, we carried out subclass mapping and the results revealed that patients in MOCS2 were more likely to respond to anti-PD1 blockades **(** **Figure 5C** **)**.

### 3.6 Single-cell analysis

**Figure 6:**
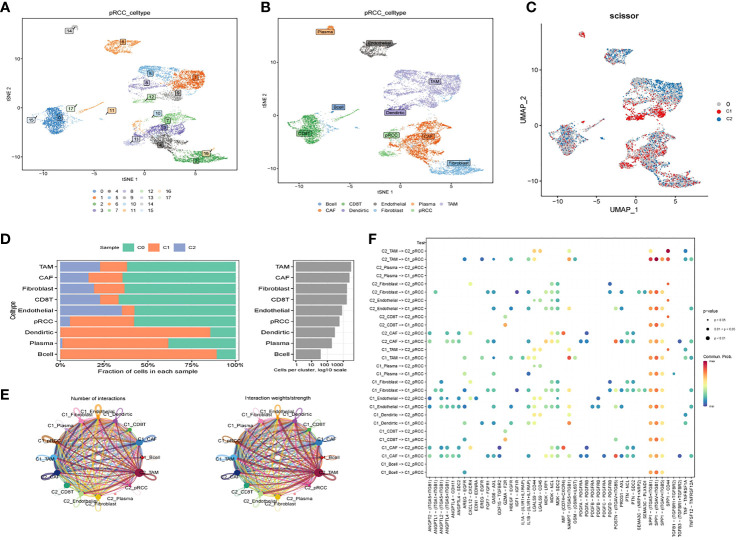
Cell–cell interactions for KIRP. **(A)** tSNE plot of the distribution of 16 samples; **(B)** tSNE plot of the distribution of nine cell clusters after clustering. **(C)** UMAP plot showing three subclusters (C0, C1, C2) of the KIRP. **(D)** The fraction of specific cell types in each cell cluster predicted by Scissor tool. **(E)** Circle plot showing the intercellular communication among major cell types in KIRP. **(F)** The ligand–receptor pairs among cells.

A total of 16 cell clusters were identified after gene filtering, normalization and principal component analysis, as shown in **Figure 6A**. There were nine specific cell types, including B cell, CD8^+^ T cell, Endothelial cell, Plasma cell, TAM cell, CAF cell, Dendritic cell, Fibroblast cell, pRCC cell **(** **Figure 6B** **)**. In addition, a total of three cell clusters (C0, C1, and C2) were predicted by Scissor tool, as shown in **Figure 6C**. The bar graph displayed the fraction of specific cell types in each cell cluster predicted by Scissor tool **(** **Figure 6D** **)**. C0 cluster was rich in TAM cell, CAF cell, Fibroblast cell, CD8+ T cell, Endothelial cell, and pRCC cell **(** **Figure 6D** **)**. C1 cluster was rich in Dendirtic cell, Plasma cell, and B cell **(** **Figure 6D** **)**. The correlation networks were generated to show the interactions among different cells **(** **Figure 6E** **)**. The ligand–receptor pairs among cells were displayed in **Figure 6F**.

## 4 Discussion

Global cancer data show that RCC accounts for about 3%~5% of adult malignant tumors, and its incidence is higher in males than in females (4, 55). RCC is the 9th most common male cancer and the 14th most common female cancer worldwide (56). As for etiology, tobacco exposure of any kind is thought to be associated with the development of kidney cancer (57). In addition, diets high in fat, high in protein, low in fruits and vegetables, and increased intake of dairy products are associated with kidney cancer, but the relative risk is not very high, and many scholars have different views (57, 58). The current diagnosis and treatment problem of renal cancer is the heterogeneity of the tumor, which often leads to different prognosis of patients with the same stage and grade (59). In addition, the incidence of tumor resistance and metastasis is high in renal cancer, and the treatment options for these patients are extremely limited, resulting in a low 5-year survival rate (60). In view of the above problems, it is necessary to determine new diagnosis and treatment strategies to improve the survival rate of patients with kidney cancer.

TCGA is an oncology research initiative of The Cancer Genome Atlas and the National Human Genome Research Institute (28). The plan includes multifactorial data on common tumor tissues and prognostic information for patients. The data included pathological sections, cancer and para-cancer transcriptome, methylation chip data and genome data (28). The development of multi-omics has made it easy for researchers to deepen their understanding of cancer at the molecular level. At the same time, a large number of omics data also brings new challenges to analysts (61). It is particularly critical to reduce data noise and obtain key characteristics of tumor occurrence and development while preserving tumor characteristics (61). Few studies have attempted to establish a comprehensive model based on multiple omics data to predict prognosis and personalized drug selection in patients with KIRP. Therefore, it is particularly important to develop a comprehensive and robust prognostic and drug selection model for patients with KIRP to assist in prognostic prediction and guide personalized treatment. In this study, we conducted a comprehensive integrated analysis of multiple omics data, including mRNA, lncRNA, miRNA, DNA methylation profile and somatic mutation data, and constructed a classifier to evaluate the prognosis of patients with KIRP and assist drug selection. Omics data are complex, multi-layered, and high weaves, so a key goal of analyzing multi-omics data is to screen for valid predictors to predict phenotypic characteristics and thus elucidate the biological significance behind them. Another major difficulty in omics data processing is dimensionality reduction, omics noise elimination and overfitting avoidance. In this study, R package Survival was first used to screen the molecular features associated with patient prognosis in each omics for subsequent analysis. The classification of cancer patients into different molecular subgroups based on multi-omics data is an important problem in the context of precision medicine. MOVICS provides a unified interface to 10 state-of-the-art multiomics ensemble clustering algorithms and integrates the downstream analyses most commonly used in cancer typing studies, including characterization and comparison of identified subtypes from multiple perspectives and validation of subtypes in external corporations of multi-class predictions using two model-free methods. Patients with KIRP were divided into three multi omics-based cancer subtypes (MOCS1, MOCS2, and MOCS3). Patients in the MOCS3 subtype were observed with shorter overall survival time than patients in the MOCS1 and MOCS2 subtypes, therefore, the classification system can be used as an important prognostic tool. Similar prognostic outcomes were observed in independent external datasets. Therefore, the classification system established by us is reliable in prognostic assessment.

In recent years, molecular typing of kidney cancer has been emerging. Molecular typing of renal cancer from genomic changes, DNA methylation profiles, RNA and protein levels has revealed repeated mutations in the PI3K/AKT pathway, suggesting that this pathway is a potential therapeutic target (62). A large number of molecular typing studies of renal cancer have emerged based on single omics or specific gene sets, recently. Chen et al. integrated multi-omics data of all kidney cancer patients based on a single algorithm but did not include data of lncRNA data in the analysis (63). Ricketts et al. integrated the multi-omics data of kidney cancer for reclassification, while this study only conducted classification from the level of each omics, without realizing the real sense of integrated multi-omics data for classification (64). Although these studies provide new directions for the diagnosis and treatment of kidney cancer to some extent, they also have certain shortcomings. The classification methods used in most typing studies are relatively simple. These shortcomings make it difficult to apply these classification studies to clinical practice. In this study, ten robust clustering algorithms based on MOVICS package were used, combined with multiple omics information, to conduct multi-omics cross-validation for patients with KIRP. Further, intra-omics heterogeneity analysis was conducted at each omics level to crack the omics differences among patients with different prognostic characteristics. Specifically, patients in the MOCS3 subtype were observed with shorter overall survival time than patients in the MOCS1 and MOCS2 subtypes. Compared to the other two subtypes, MOCS1was negatively correlated with immune-related pathways. Global dysfunction of cancer-related pathways among the three MOCS subtypes were also observed. We also evaluated the immune microenvironmental cell abundance and immune checkpoint and compared the discrepancy among the MOCSs.

Our study unexpectedly found that these three MOCSs also have significant differences in sensitivity to molecularly targeted drugs. We observed that patients in MOCS3 were more sensitive to Crizotinib, Erlotinib, Pazopanib, Saracatinib, Sunitinib, and Temsirolimus; while patients in MOCS1 were more sensitive to AS601245, Bosutinib, PAC.1, ABT.888, and Bleomycin. Whereafter, the results of subclass mapping revealed that patients in MOCS2 were more likely to respond to anti-PD1 blockades. In recent years, the treatment of kidney cancer has evolved from non-specific immune approaches to targeted therapy of vascular endothelial growth factor (VEGF), and now to novel immunotherapies. Our study assessed therapeutic differences among different subtypes and therefore can be a potential therapeutic direction for patients with KIRP.

In summary, our study provides a new reference for molecular subtypes of KIRP risk. In this study, a robust prognostic and drug selection subtype system was constructed by integrating multiple omics data using multiple algorithms. However, there are still some limitations in our study. Firstly, multi-omics data used for molecular subtypes is difficult to be applied in clinical practice. Second, although we compared the enrichment pathway and drug sensitivity between subgroups, further experiments and external data sets are still needed for verification.

## Data availability statement

The original contributions presented in the study are included in the article/**Supplementary Material**. Further inquiries can be directed to the corresponding author.

## Author contributions

RL designed the study. BW and ML performed data analysis. BW drafted the manuscript. RL revised the manuscript. All authors contributed to the article and approved the submitted version.

## Supplementary material

Supplementary Figure 1 Identifying and verifying of the multi omics-based cancer subtype. **(A)** Quantification of sample similarity using silhouette score based on the consensus ensembles result. **(B)** Three MOCSs were also identified as predicted by the external GSE2748 cohort. **(C)** Comparison of the overall survival time for the three MOCSs.

Supplementary Figure 2 Functional enrichment analysis of MOCS1, MOCS2 and MOCS3 subgroups. **(A)** Heatmap of metabolism-related enrichment scores among the three MOCSs. **(B)** Heatmap of immune-related enrichment scores among the three MOCSs.

Supplementary Figure 3 The landscapes of specific immune scores among the three MOCSs. **(A)** Comparison of the signature score among the three MOCSs. **(B)** Comparison of the RNAss, DNAss, DMPss, ENHss, EREG.EXPss, and HRD among the three MOCSs.

Supplementary Figure 4 Landscapes of somatic mutations and potential targets in the two subtypes. **(A)** Waterfall plot showing the mutation patterns of the top 20 most frequently mutated genes among the three MOCSs. **(B)** The synthetic lethal mutations in MOCS1, MOCS2, and MOCS3. **(C)** Potential druggable gene categories from the mutation dataset for MOCS1, MOCS2, and MOCS3. **(D)** The fraction of pathways or samples of oncogenic signaling pathways for MOCS1, MOCS2, and MOCS3.
