# Supplementary figures and images for: Identification and verification of prognostic cancer subtype based on multi-omics analysis for kidney renal papillary cell carcinoma
Source: Front Oncol. 2023 Apr 5;13:1169395. doi: 10.3389/fonc.2023.1169395 (PMC10113630; doi:10.3389/fonc.2023.1169395)

Figure S1

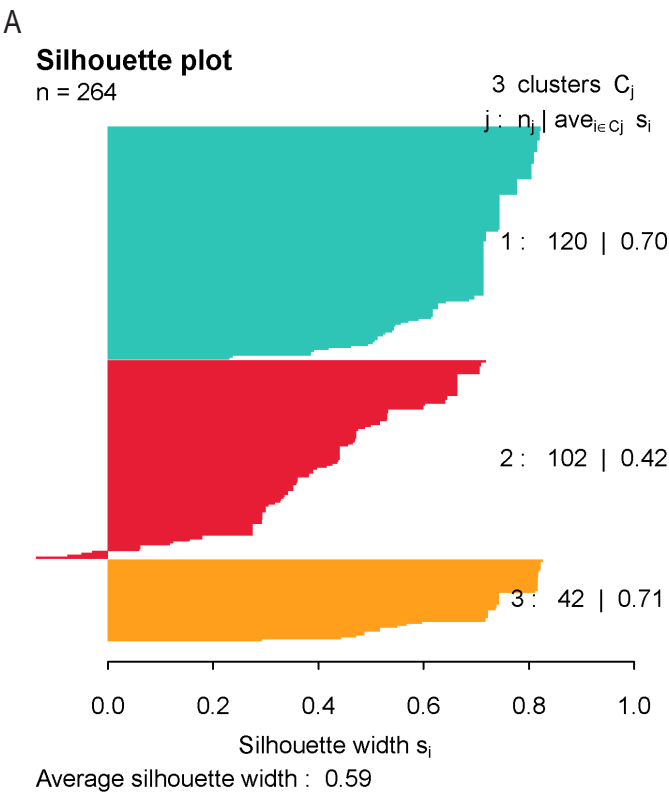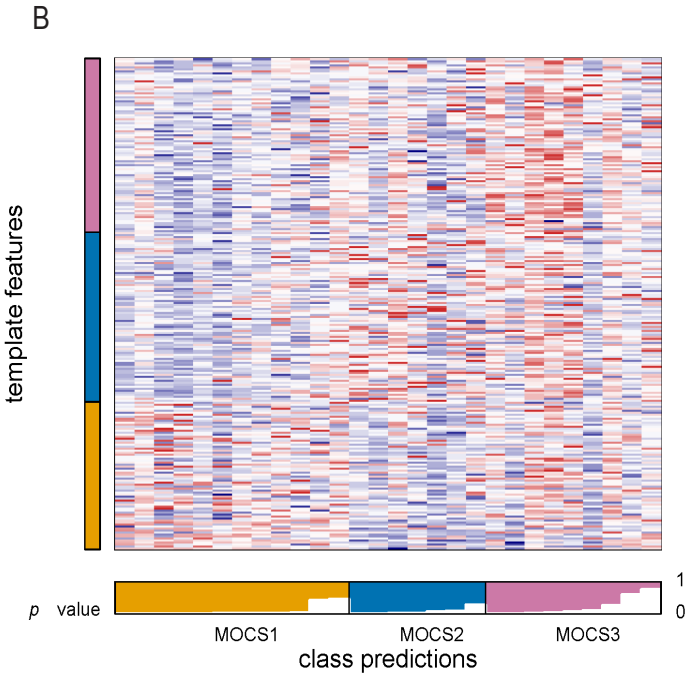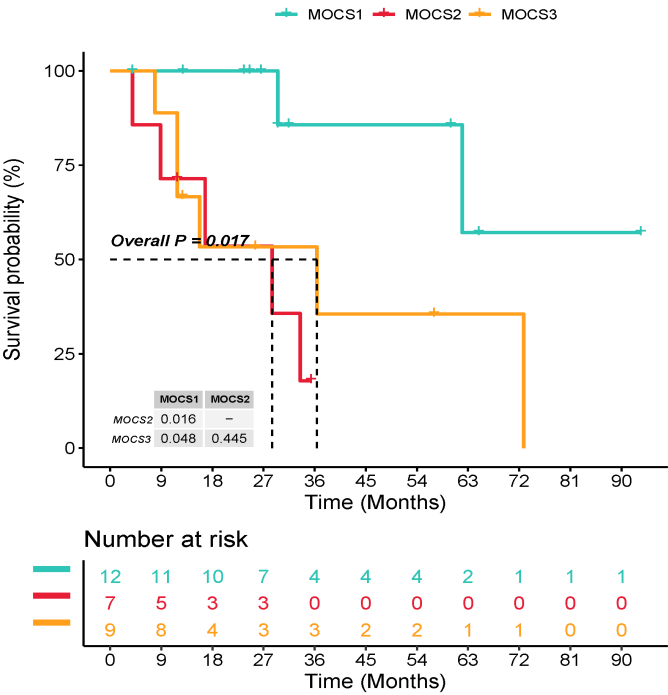

Supplement: Supplementary Figure 1 — Identifying and verifying of the multi omics-based cancer subtype. (A) Quantification of sample similarity using silhouette score based on the consensus ensembles result. (B) Three MOCSs were also identified as predicted by the external GSE2748 cohort. (C) Comparison of the overall survival time for the three MOCSs. [file Image_1.pdf]

Figure S2

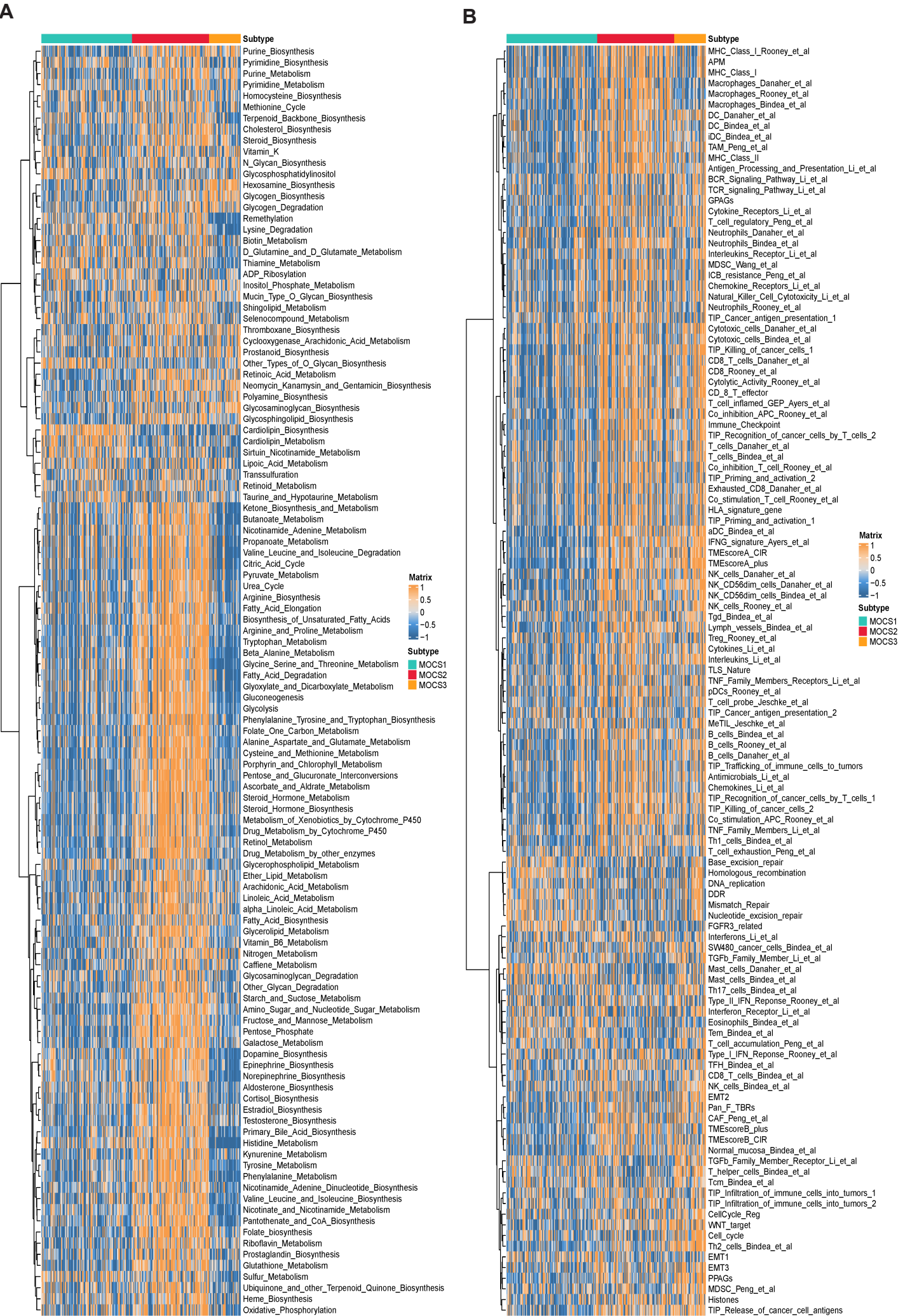

Supplement: Supplementary Figure 2 — Functional enrichment analysis of MOCS1, MOCS2 and MOCS3 subgroups. (A) Heatmap of metabolism-related enrichment scores among the three MOCSs. (B) Heatmap of immune-related enrichment scores among the three MOCSs. [file Image_2.pdf]

Figure S3

A

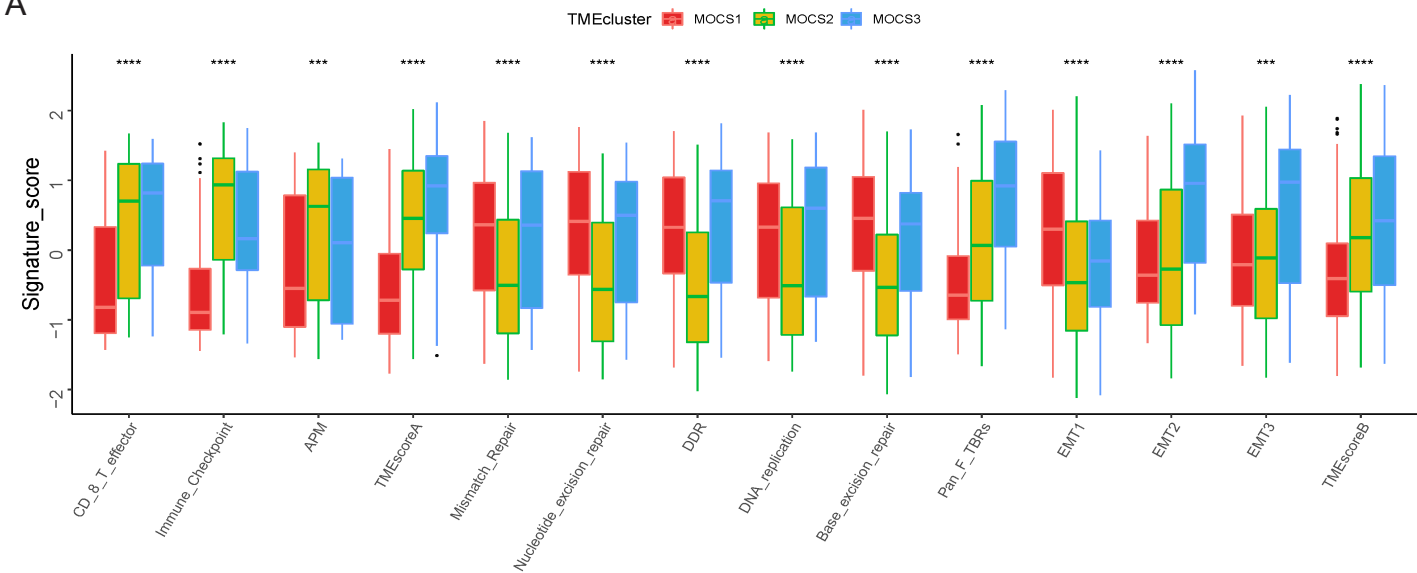

B

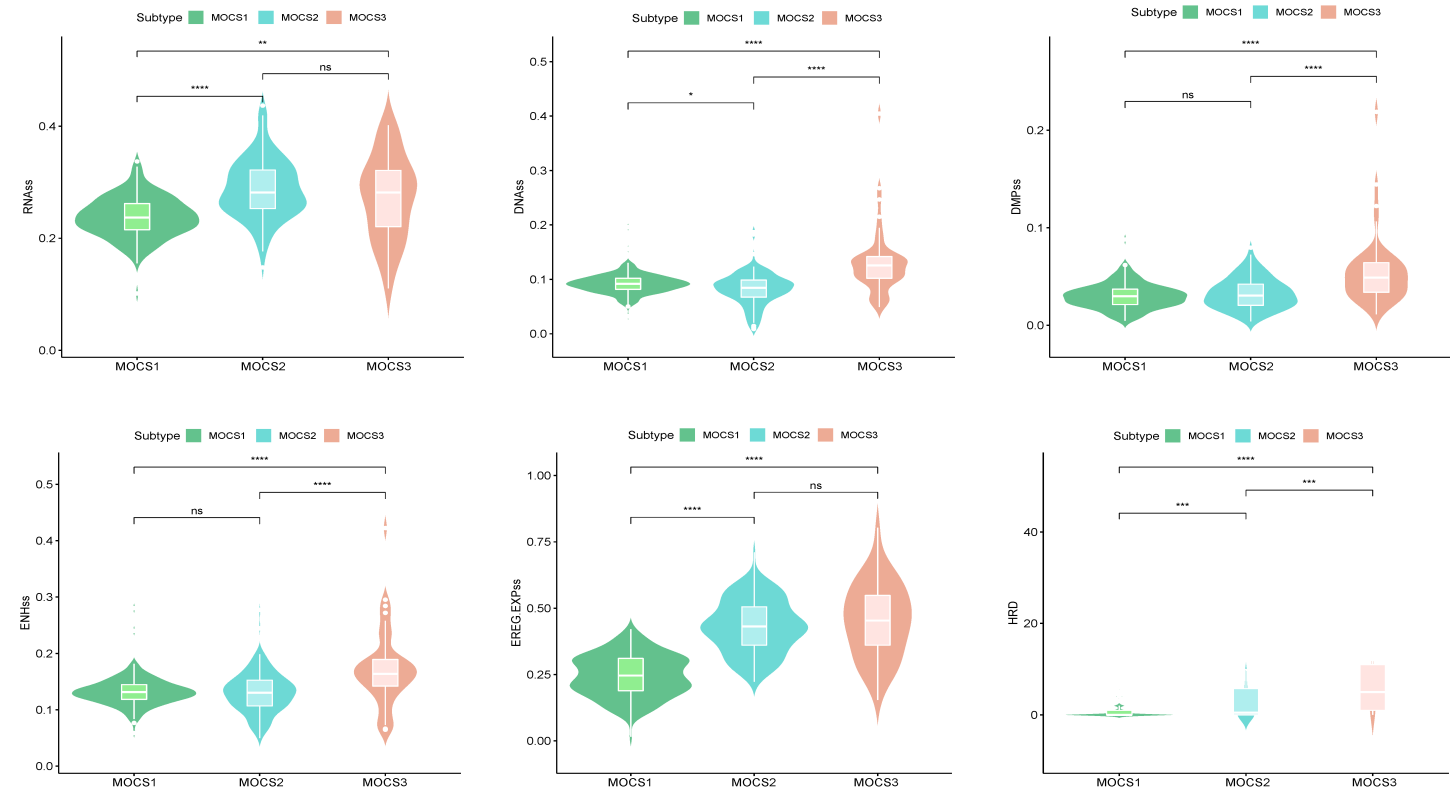

Supplement: Supplementary Figure 3 — The landscapes of specific immune scores among the three MOCSs. (A) Comparison of the signature score among the three MOCSs. (B) Comparison of the RNAss, DNAss, DMPss, ENHss, EREG.EXPss, and HRD among the three MOCSs. [file Image_3.pdf]

Figure S4

A

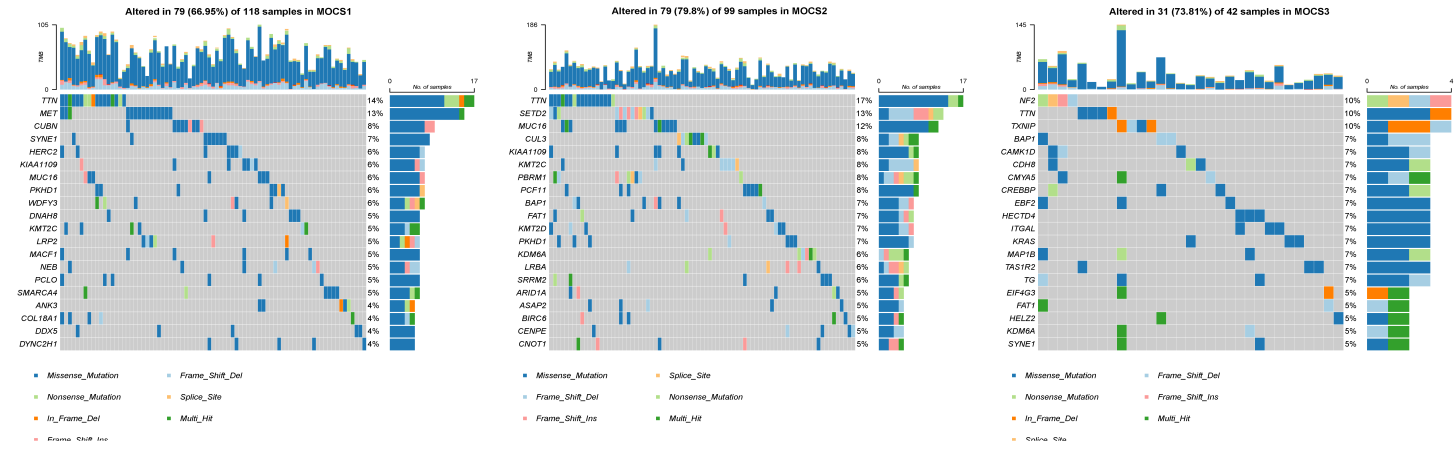

B

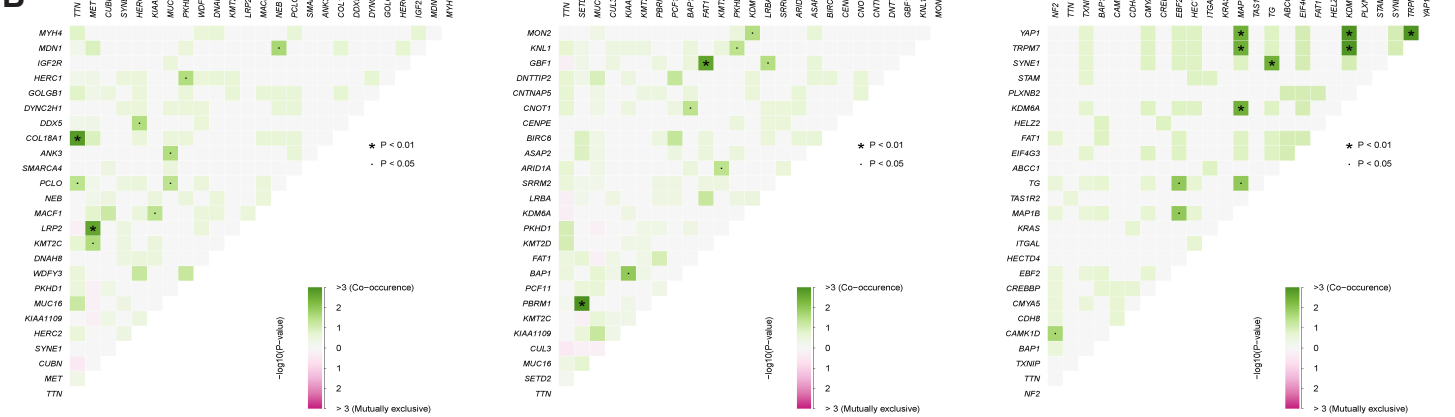

C

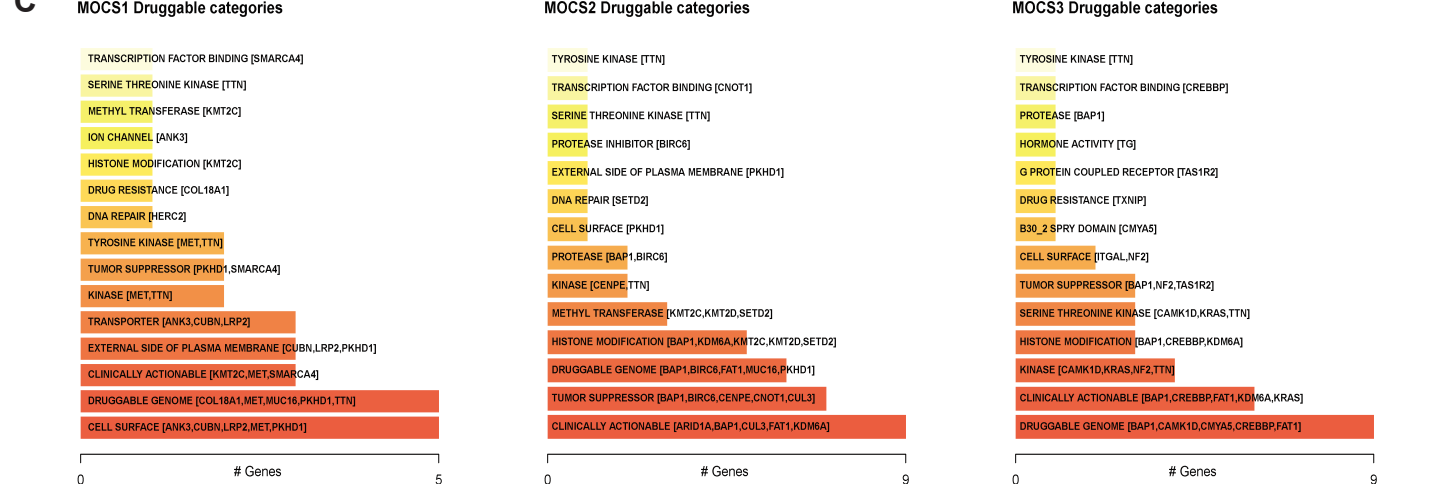

D

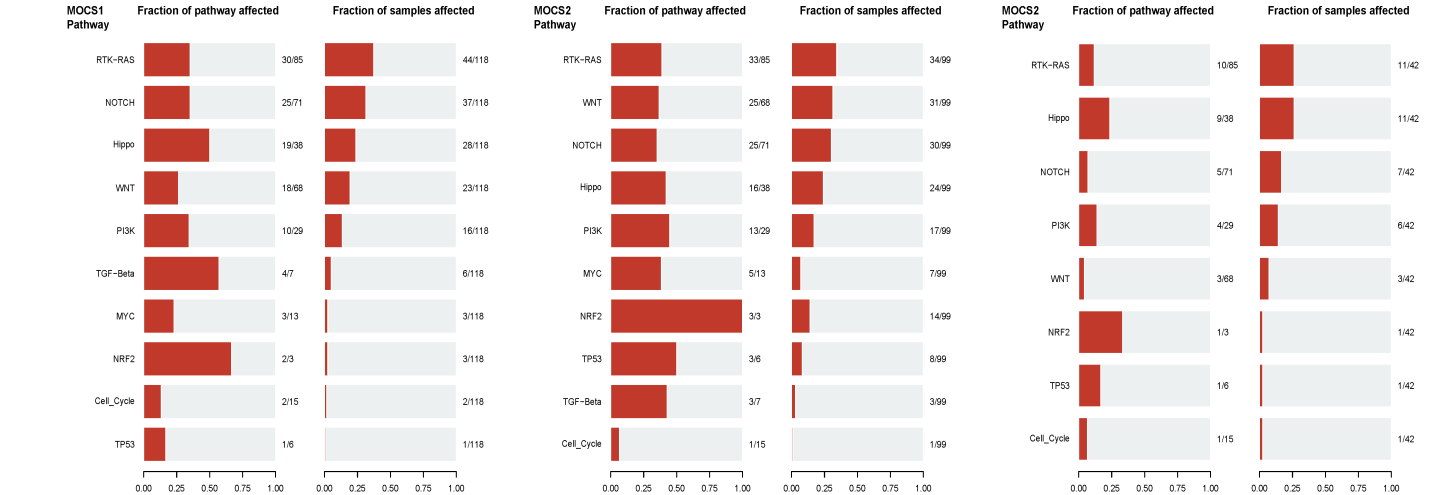

Supplement: Supplementary Figure 4 — Landscapes of somatic mutations and potential targets in the two subtypes. (A) Waterfall plot showing the mutation patterns of the top 20 most frequently mutated genes among the three MOCSs. (B) The synthetic lethal mutations in MOCS1, MOCS2, and MOCS3. (C) Potential druggable gene categories from the mutation dataset for MOCS1, MOCS2, and MOCS3. (D) The fraction of pathways or samples of oncogenic signaling pathways for MOCS1, MOCS2, and MOCS3. [file Image_4.pdf]
