# Supplementary material for: Identification and verification of prognostic cancer subtype based on multi-omics analysis for kidney renal papillary cell carcinoma
Source: Front Oncol. 2023 Apr 5;13:1169395. doi: 10.3389/fonc.2023.1169395 (PMC10113630; doi:10.3389/fonc.2023.1169395)
Supplement: Supplementary file 5 [file DataSheet_1.docx]

rm(list = ls())

exp <- read.csv("TCGA.COAD.exp.csv",row.names = 1)

surv <- read.csv("TCGA.COAD.surv.csv",row.names = 1)

clin <- read.csv("TCGA.COAD.clin.csv",row.names = 1)

DEG1 <- read.csv("CRC.DEG1.csv",row.names = 1)

library(reshape2)

library(ggpubr)

library(limma)

library(GSEABase)

library(GSVA)

library(org.Hs.eg.db)

library(clusterProfiler)

diff <- rownames(DEG1)

diff_entrez <- bitr(diff,

fromType = "SYMBOL",

toType = "ENTREZID",

OrgDb = "org.Hs.eg.db")

go.diff <- enrichGO(gene = diff_entrez$ENTREZID,

OrgDb = org.Hs.eg.db,

pAdjustMethod = 'BH',

pvalueCutoff =0.01,

qvalueCutoff = 0.05,

ont="all",

readable =T)

GO_result <- go.diff@result

#write.csv(GO_result,"GO_result.COAD.1.csv")

GO_result <- GO_result[order(GO_result$ONTOLOGY, GO_result$pvalue), ]

GO_result$Description <- factor(GO_result$Description,

levels = GO_result$Description)

ggplot(GO_result, aes(Description, -log10(pvalue))) +

geom_col(aes(fill = ONTOLOGY), width = 0.5) +

scale_fill_manual(values = c('#8EA1CB', '#67C1A5', '#FA8E61')) +

scale_y_continuous(expand = expansion(mult = c(0, 0.1))) +

coord_flip() +

labs(x = '', y = '-Log10 P-Value\n')+

facet_grid(ONTOLOGY~., scale = 'free_y', space = 'free_y') +

theme(panel.grid = element_blank(), panel.background = element_rect(color = 'black', size = 1,

fill = 'transparent')) +

theme(legend.position = 'none')

KEGG_diff <- enrichKEGG(gene = diff_entrez$ENTREZID,

organism = "hsa",#物种，Homo sapiens (human)

pvalueCutoff = 0.05,

qvalueCutoff = 0.05)

KEGG_result <- KEGG_diff@result

View(KEGG_result)

#write.csv(KEGG_result,"KEGG_result.COAD.1.csv")

go4 <- KEGG_result[1:20,]

library(ggsci)

ggplot(go4,aes(Description,Count))+

geom_point(aes(size = Count,

fill = -log10(pvalue)),

alpha = 0.7,

color = "black",

pch=21)+

theme_test()+

coord_flip()+

scale_fill_distiller(palette = "YlBu",direction = 1) +

theme(axis.text.x = element_text(size = 10))+

theme(legend.position = "left")+

xlab('')

library(ggplot2)

library(tibble)

library(ggsci)

library(glmnet)

library(survival)

library(ggprism)

library(dplyr)

DEG2 <- DEG1[DEG1$p_val_adj <0.05,]

exp1 <- t(exp)

exp1 <- exp1[rownames(exp1) %in% rownames(DEG2),]

exp1 <- t(exp1)

data <- cbind(exp1,surv)

ntree <- 500

spe_rf <- randomSurvivalForest::rsf(Surv(time,event) ~ ., data = data, ntree = ntree,mtry=3,nodesize = 3,splitrule = "logrank",seed = 3,importance = TRUE)

spe_rf

randomSurvivalForest::plot.error(spe_rf)

raw.imp <- spe_rf$importance;

rel.imp <- raw.imp/max(abs(raw.imp))

imp.res <- data.frame(gene = names(raw.imp),

raw.importance = raw.imp,

rel.importance = rel.imp,

stringsAsFactors = F)

imp.cutoff <- 0

rel.imp.sel <- rel.imp[rel.imp > imp.cutoff] # 取出大于阈值的变量

rel.imp.sel <- sort(rel.imp.sel,decreasing = T) # 根据重要性排序

rel.imp.sel <- as.data.frame(rel.imp.sel)

rel.imp.sel$gene <- rownames(rel.imp.sel)

library(ggplot2)

library(ggpubr)

rel.imp.sel <- rel.imp.sel[rel.imp.sel$gene %in% c("UBASH3A","ARL3","TPGS1","CDKN2A","IFITM10"),]

ggplot(rel.imp.sel) +

geom_segment(aes(x=gene, xend=gene, y=0,yend=rel.imp.sel,

color = rel.imp.sel),alpha = 0.9,size=9) +

geom_hline(yintercept = 0.5,lty=2,color="#38493E")+

scale_x_discrete(limits = rev(rel.imp.sel$gene)) +

scale_color_distiller(palette = "RdPu",direction = 1) +

labs(x=NULL, y = "Relative Importance") +

theme_classic() +

coord_flip()+

theme(panel.grid = element_blank(),

axis.text.x = element_text(size = 10, color = "black"),

axis.text.y = element_text(size = 12, color = "black")) +

theme(legend.position = "none")

exp5 <- exp[,colnames(exp) %in% c("UBASH3A","ARL3","TPGS1","CDKN2A","IFITM10")]

library(survival)

library(survminer)

model = coxph(formula = Surv(time, event) ~ UBASH3A + TPGS1 + CDKN2A + IFITM10 + ARL3, data = data)

coef(model)

surv$riskscore = as.numeric(predict(model,type="risk",data))

riskscore <- surv$riskscore

ri = ifelse(riskscore < median(riskscore),"lowrisk","highrisk")

names(ri) = names(riskscore)

ri = factor(ri,levels = c("lowrisk","highrisk"))

table(ri)

data$ri = ri

sfit <- survfit(Surv(time,event)~ri, data=surv)

ggsurvplot(sfit,pval = T,

surv.median.line = "hv",

xlab = "Overall survival",

censor.shape = 124,censor.size = 3,

risk.table = TRUE,

tables.height = 0.25,

conf.int = TRUE,

palette = c("steelblue3","darksalmon"),

ggtheme = theme_pubr())

fp_dat=data.frame(patientid=1:length(riskscore),

riskscore=as.numeric(sort(riskscore)),

ri = ri[order(riskscore)])

datarGEO$riskscore <- pdr$riskscore

datarGEO <- datarGEO[order(datarGEO$riskscore),]

sur_dat=data.frame(patientid=1:length(riskscore),

time=datarGEO$time,

event=datarGEO$event)

sur_dat$event=ifelse(sur_dat$event==0,'alive','death')

sur_dat$event=factor(sur_dat$event,levels = c("death","alive"))

fp_dat=data.frame(patientid=1:length(riskscore),

riskscore=as.numeric(sort(riskscore)),

ri = ri[order(riskscore)])

ggplot(fp_dat,aes(x=patientid,y=riskscore,color = ri))+

geom_point(alpha = 0.8,size = 1)+

scale_color_manual(values = c("steelblue3","darksalmon"))+

geom_vline(xintercept = sum(riskscore<median(riskscore)),lty = 2)+

scale_x_continuous(expand=c(0,0))+

theme_classic()

ggplot(sur_dat,aes(x=patientid,y=time))+

geom_point(aes(col=event),alpha = 0.8 ,size =1)+

scale_color_manual(values = c("darksalmon","steelblue3"))+

geom_vline(xintercept = sum(riskscore<median(riskscore)),lty = 2)+

scale_x_continuous(expand=c(0,0))+

theme_classic()

library(reshape2)

library(ggpubr)

library(limma)

library(GSEABase)

library(GSVA)

gmtFile="immune.gmt"

geneSets=getGmt(gmtFile, geneIdType=SymbolIdentifier())

ssgseaScore=gsva(t(exp), geneSets, method='ssgsea', kcdf='Gaussian', abs.ranking=TRUE)

ssgseaOut=rbind(id=colnames(ssgseaScore), ssgseaScore)

ssgseaOut = ssgseaOut[-1,]

ssgseaOut1 <- t(ssgseaOut)

ssgseaOut1 <- as.data.frame(ssgseaOut1)

write.csv(ssgseaOut1,"ssgseaOut_CRC.1.csv")

ssgseaOut2 <- read.csv("ssgseaOut_CRC.1.csv",row.names = 1)

colnames(ssgseaOut2) <- gsub("\\."," ",colnames(ssgseaOut2))

library(Rmisc)

library(corrplot)

library(ggcorrplot)

corr <- cor(ssgseaOut2, method = "spearman")

env.p <- cor_pmat(ssgseaOut2,method = "spearman")

library(corrplot)

corrplot(corr,title = "",

method = "circle", #或"circle" (default), "square", "ellipse", "number", "pie", "shade" and "color"

outline = T, addgrid.col = "darkgray",

type = "upper",tl.pos="lt",

tl.col = "black")

corrplot(corr,type="lower",add=TRUE,method="number",

tl.pos="n",tl.cex=1.2,diag=FALSE, cl.pos="n",

number.cex = 0.4)

ssgseaOut3 <- cbind(surv,ssgseaOut2)

ssgseaOut3 <- ssgseaOut3[,-(1:3)]

ssgseaOut3$ri <- data$ri

ssgseaOut3 <- as.data.frame(ssgseaOut3)

ssgseaOut3$ID = rownames(ssgseaOut3)

ssgsea_barplot <- ssgseaOut3 %>%

gather(key = Cell_type,value = Score,1:23)

library(ggsci)

ggplot(ssgsea_barplot, aes(x= Cell_type, y= Score, color= ri)) +

stat_boxplot(geom = "errorbar",width = 0.5)+

geom_boxplot(width = 0.5,

size = 0.5,

alpha = 1) +

theme_classic() +

coord_flip()+

labs(x = "", y = "ssGSEA",color = "risk") +

scale_color_npg() +

theme(axis.text.x = element_text(angle = 45,hjust=1))+

stat_compare_means(aes(group=ri),

method="wilcox.test",

symnum.args=list(cutpoints=c(0, 0.001, 0.01, 0.05, 1), symbols=c("***", "**", "*", "")), label="p.signif")

sca <- cbind(t(ssgseaOut),exp5)

sca <- as.data.frame(sca)

sca$riskscore <- data$riskscore

write.csv(sca,"1.24.linshi.csv")

sca <- read.csv("1.24.linshi.csv",row.names = 1)

sca2 <- t(sca)

library(ggpubr)

library(ggExtra)

p1 <- sca%>%ggplot(aes(x = riskscore, y = `T.follicular.helper.cell`))+

ylab("T follicular helper cell")+

geom_point(size=2,alpha=0.2,color="grey20")+

geom_smooth(method = "lm", formula = y~x, color = "#756bb1", fill = "#cbc9e2")+

theme_bw()+

stat_cor()

ggMarginal(p1, type = "density", xparams = list(fill = "#FFE4B5"),yparams = list(fill = "steelblue3"))

myro <- cbind(ssgseaOut2,exp5)

library(psych)

spearman <- corr.test(myro[,1:23], myro[,24:28], method = 'spearman', adjust = 'none')

library(reshape2)

p <- data.frame(spearman$p)

p$myro <- rownames(p)

p <- melt(p, id = 'myro')

spearman1 <- cbind(p)

r <- data.frame(spearman$r)

r$myro <- rownames(r)

r <- melt(r, id = 'myro')

spearman <- cbind(r)

spearman$p <- spearman1$value

mark <- matrix(case_when(spearman$p < 0.001~"***",

spearman$p < 0.01~"**",

spearman$p< 0.05~"*",

T~""),

nrow = nrow(spearman))

spearman$mark <- mark

spearman$value <- round(spearman$value,2)

my_palette <- colorRampPalette(c('steelblue4', 'white', 'red4'), alpha=TRUE)(n=128)

ggplot(data = spearman, aes(x = variable, y = myro, fill = value)) +

geom_tile(aes(fill= value),color = "black") +

geom_text(aes(label = mark),color = "black",size = 3)+

geom_text(aes(label = value),color = "black",size = 2,vjust = 1.8)+

scale_fill_gradientn(colors = c('gold3', 'white', 'darkseagreen4')) +

labs(y = '', x = '', color = '-log10Pvalue')+

theme_classic()+

theme(axis.text.x = element_text(size=9,angle = 45,hjust = 1,color = "black"),

axis.text.y = element_text(size=10,color = "black"),

panel.background=element_blank())

input_data <- ssgseaOut2

library(vegan)

library(dplyr)

library(linkET)

library(corrplot)

mantel <- mantel_test(spec = exp5, env = input_data,

spec_select = list(Gene = 1:5),

mantel_fun = "mantel") %>%

mutate(rd = cut(r, breaks = c(-Inf, 0.2, 0.4, Inf),

labels = c("< 0.2", "0.2 - 0.4", ">= 0.4")),

pd = cut(p, breaks = c(-Inf, 0.01, 0.05, Inf),

labels = c("< 0.01", "0.01 - 0.05", ">= 0.05")))

mantel

qcorrplot(correlate(input_data), type = "full", diag = FALSE) +

geom_square() +

geom_couple(aes(colour = pd, size = rd), data = mantel, curvature = 0.1) +

scale_fill_gradientn(colours = RColorBrewer::brewer.pal(11, "PRGn")) +

scale_size_manual(values = c(0.5, 1, 2)) +

scale_colour_manual(values = color_pal(3)) +

guides(size = guide_legend(title = "Mantel's r",

override.aes = list(colour = "grey35"),

order = 2),

colour = guide_legend(title = "Mantel's p",

override.aes = list(size = 3),

order = 1),

fill = guide_colorbar(title = "Pearson's r", order = 3))

library(xCell)

library(tidyr)

xCell <- xCellAnalysis(t(exp))

xcell1 <- t(xCell)

xcell1 <- as.data.frame(xcell1)

xcell1$group = data$ri

xcell1$ID = rownames(xcell1)

xcell1_barplot <- xcell1 %>%

gather(key = Cell_type,value = Score,1:67)

library(ggsci)

library(ggplot2)

library(reshape2)

ggplot(xcell1_barplot[14785:30016,],aes(x = Cell_type , y = Score, color = group)) +

stat_boxplot(geom = "errorbar",width = 0.5)+

geom_boxplot(outlier.size = 1,width = 0.5,size = 0.5) +

theme_classic() +

labs(x = "xCell", y = "") +

scale_color_npg()+

ylim(c(0,0.4))+

theme(axis.text.x = element_text(size = 7,angle = 45,hjust = 1)) +

stat_compare_means(aes(group=group),

method="wilcox.test",

symnum.args=list(cutpoints=c(0, 0.001, 0.01, 0.05, 1), symbols=c("***", "**", "*", "ns")), label="p.signif",hide.ns = T)

rb1 <- t(xCell)

rb1 <- as.data.frame(rb1)

rb1$riskscore <- data$riskscore

rb2 <- t(rb1)

p1 <- rb1%>%ggplot(aes(x = riskscore, y = `Mast cells`))+

geom_point(size=2,alpha=0.2,color="grey20")+

geom_smooth(method = "lm", formula = y~x, color = "#756bb1", fill = "#cbc9e2")+

theme_bw()+

stat_cor()

ggMarginal(p1, type = "density", xparams = list(fill = "#FFE4B5"),yparams = list(fill = "steelblue3"))

library(estimate)

exp <- t(exp)

write.table(exp,file = "TCGA.COAD.exp.txt",sep = "\t",quote = F)

filterCommonGenes(input.f = "TCGA.COAD.exp.txt",

output.f="OV_10412genes.gct",

id="GeneSymbol")

estimateScore(input.ds = "OV_10412genes.gct",

output.ds="OV_estimate_score.gct",

platform="affymetrix")

plotPurity(scores="OV_estimate_score.gct", samples="s516",

platform="affymetrix")

scores=read.table("OV_estimate_score.gct",skip = 2,header = T)

rownames(scores)=scores[,1]

scores=t(scores[,3:ncol(scores)])

write.csv(scores,"estimate.CRC.1.csv")

estimate1 <- scores

estimate1 <- as.data.frame(estimate1)

estimate1$risk = data$ri

estimate1$riskscore = data$riskscore

estimate1 <- estimate1[,-4]

library(ggpubr)

estimate_barplot <- estimate1 %>%

gather(key = Cell_type,value = Score,1:3)

ggplot(estimate_barplot,

aes(x = Cell_type,y = Score,

fill = risk,color = risk)) +

stat_boxplot(geom = "errorbar",width = 0.2,

position=position_dodge(0.9))+

geom_violin(width = 0.9,alpha = 0.5) +

geom_boxplot(width=0.2,fill = "white",

position=position_dodge(0.9))+

theme_classic() +

scale_fill_aaas()+

scale_color_aaas()+

labs(x = "", y = "") +

annotate("text",label="",size=3.5,x=1.5,y=1500)+

stat_compare_means(aes(group=risk),

method="t.test",

symnum.args=list(cutpoints=c(0, 0.001, 0.01, 0.05, 1), symbols=c("***", "**", "*", "ns")), label="p.signif",hide.ns = T)

p1 <- estimate1%>%ggplot(aes(x = riskscore, y = ESTIMATEScore))+

geom_point(size=2,alpha=0.2,color="grey20")+

geom_smooth(method = "lm", formula = y~x, color = "#756bb1", fill = "#cbc9e2")+

theme_bw()+

stat_cor()

ggMarginal(p1, type = "density", xparams = list(fill = "#FFE4B5"),yparams = list(fill = "steelblue3"))

estimate <- estimate[,-4]

myro <- cbind(estimate,exp5)

library(psych)

spearman <- corr.test(myro[,1:3], myro[,4:8], method = 'spearman', adjust = 'none')

library(reshape2)

p <- data.frame(spearman$p)

p$myro <- rownames(p)

p <- melt(p, id = 'myro')

spearman1 <- cbind(p)

r <- data.frame(spearman$r)

r$myro <- rownames(r)

r <- melt(r, id = 'myro')

spearman <- cbind(r)

spearman$p <- spearman1$value

mark <- matrix(case_when(spearman$p < 0.001~"***",

spearman$p < 0.01~"**",

spearman$p< 0.05~"*",

T~""),

nrow = nrow(spearman))

spearman$mark <- mark

spearman$value <- round(spearman$value,2)

my_palette <- colorRampPalette(c('steelblue4', 'white', 'red4'), alpha=TRUE)(n=128)

ggplot(data = spearman, aes(x = variable, y = myro, fill = value)) +

geom_tile(aes(fill= value),color = "black") +

geom_text(aes(label = mark),color = "black",size = 3)+

geom_text(aes(label = value),color = "black",size = 2,vjust = 1.8)+

scale_fill_gradientn(colors = c('gold3', 'white', 'darkseagreen4')) +

labs(y = '', x = '', color = '-log10Pvalue')+

theme_classic()+

theme(axis.text.x = element_text(size=9,angle = 45,hjust = 1,color = "black"),

axis.text.y = element_text(size=10,color = "black"),

panel.background=element_blank())

#基因环形图

library(RCircos)

library(magrittr)

library(tidyverse)

library(rtracklayer)

library(TCGAbiolinks)

genes <- colnames(exp5)

gene_pos <- import("hg38.gtf") %>% # 载入gtf文件

as.data.frame %>%

# 仅选择基因，去除转录本等等

filter(source == "HAVANA", type == "gene") %>%

# 保留基因位置和名称

dplyr::select(seqnames, start, end, gene_name) %>%

# 挑选目的基因

filter(gene_name %in% genes)

head(gene_pos)

# 原文没说内圈散点图代表什么特征，我们这里随机生成一列数值

gene_pos$gene_dot <- rnorm(nrow(gene_pos), 0, 2)

# 保存到文件，便于套用格式

write.csv(gene_pos,"ye_easy_input.csv", row.names = F, quote = F)

# 加载基因所在的位置和数值

gene_pos <- read.csv("ye_easy_input.csv", header = T)

# 加载染色体Ideogram

(data("UCSC.HG38.Human.CytoBandIdeogram"))

# 根据hg38构建染色体位置，只保留chr1-22,X,Y，在圈内部构建三圈轨道

RCircos.Set.Core.Components(UCSC.HG38.Human.CytoBandIdeogram,

chr.exclude = NULL,

tracks.inside = 3,

tracks.outside = 0)

RCircos.Set.Plot.Area()

# 绘制染色体

RCircos.Chromosome.Ideogram.Plot()

# 在第一圈用散点在基因所在的位置标注数值

# 调整配色

params <- RCircos.Get.Plot.Parameters()

params$track.background <- "grey" # 第三圈默认配色为wheat，模仿原文修改为灰色

RCircos.Reset.Plot.Parameters(params)

RCircos.Scatter.Plot(gene_pos,

data.col = 5, # 用第5列的数值作为点的纵坐标

by.fold = 1, # 点的颜色cutoff，大于等于1的基因显示为红色点，小于等于-1的显示为蓝色点，-1到1之间为黑点

track.num = 1,

side = "in")

# 在第二圈绘制线段标注基因所在的位置

RCircos.Gene.Connector.Plot(genomic.data = gene_pos,

track.num = 2,

side = "in")

# 在第三圈标注基因名

RCircos.Gene.Name.Plot(gene_pos,

name.col = 4,

track.num = 3,

side = "in")

corr <- cor(exp5, method = "spearman")

env.p <- cor_pmat(exp5,method = "spearman")

corrplot(corr,title = "",

method = "circle", #或"circle" (default), "square", "ellipse", "number", "pie", "shade" and "color"

outline = T, addgrid.col = "darkgray",

type = "upper",tl.pos="lt",

tl.col = "black")

corrplot(corr,type="lower",add=TRUE,method="number",

tl.pos="n",tl.cex=1.2,diag=FALSE, cl.pos="n",

number.cex = 1)

View(clin)

clin$riskscore <- data$riskscore

clin$event <- data$event

clin$time <- data$time

clin <- na.omit(clin)

library(survival)

library(regplot)

library(rms)

clin2 <- clin[,-(1:3)]

clin2 <- clin2[!clin2$Stage %in% c("not reported"),]

res.cox=coxph(Surv(time, event) ~ . , data = clin2)

nom1=regplot(res.cox,

plots = c("density", "boxes"),

clickable=F,

title="",

points=TRUE,

droplines=TRUE,

rank="sd",

dencol = "lightblue",

boxcol = "lightblue",

failtime = c(12,36,60),

prfail = F)

exp5 <- as.data.frame(exp5)

exp5$risk = data$ri

exp5_barplot <- exp5 %>%

gather(key = Cell_type,value = Score,1:5)

library(ggsci)

library(ggplot2)

library(reshape2)

ggplot(exp5_barplot,aes(x = Cell_type , y = Score,

color = risk,)) +

stat_boxplot(geom = "errorbar",width = 0.5)+

geom_boxplot(outlier.size = 1,width = 0.5,size = 0.5) +

theme_bw() +

coord_flip()+

scale_color_jama()+

labs(x="",y="")+

theme(axis.text.x = element_text(size = 10)) +

stat_compare_means(aes(group=risk),

method="wilcox.test",

symnum.args=list(cutpoints=c(0, 0.001, 0.01, 0.05, 1), symbols=c("***", "**", "*", "ns")), label="p.signif",hide.ns = T)

ggforest(model,data)

age2 = ifelse(clin2$Age < 50,"<50",">=50")

clin2$age2 <- age2

ggplot(clin2,

aes(x = age2,y = riskscore,

fill = age2,color = age2)) +

stat_boxplot(geom = "errorbar",width = 0.2,

position=position_dodge(0.9))+

geom_violin(width = 0.9,alpha = 0.5) +

geom_boxplot(width=0.2,fill = "white",

position=position_dodge(0.9))+

theme_classic() +

scale_fill_aaas()+

scale_color_aaas()+

ylim(c(0,5))+

labs(x = "", y = "",color = "Age",fill = "Age") +

annotate("text",label="",size=3.5,x=1.5,y=1500)+

stat_compare_means(aes(group=age2),

method="wilcox.test",

symnum.args=list(cutpoints=c(0, 0.001, 0.01, 0.05, 1), symbols=c("***", "**", "*", "ns")), label="p.signif",hide.ns = T)

library(TCGAbiolinks)

library(maftools)

query <- GDCquery(

project = "TCGA-COAD",

data.category = "Simple Nucleotide Variation",

data.type = "Masked Somatic Mutation",

access = "open"

)

GDCdownload(query)

GDCprepare(query, save = T,save.filename = "TCGA-COAD_SNP.Rdata")

surv$ri = ri

library(maftools)

load(file = "TCGA-COAD_SNP.Rdata")

data$Tumor_Sample_Barcode <- str_sub(data$Tumor_Sample_Barcode,1,16)

library(stringr)

data <- data[data$Tumor_Sample_Barcode %in% rownames(surv),]

surv_low <- surv[surv$ri %in% c("highrisk"),]

data_low <- data[data$Tumor_Sample_Barcode %in% rownames(surv_low),]

maf.coad <- data_low

class(maf.coad)

dim(maf.coad)

maf.coad[1:10,1:10]

maf <- read.maf(maf.coad)

plotmafSummary(maf = maf, rmOutlier = TRUE, addStat = 'median',dashboard = TRUE,color = vc_cols)

library(RColorBrewer)

vc_cols <- brewer.pal(8,"Dark2")

names(vc_cols) = c(

'Frame_Shift_Del',

'Missense_Mutation',

'Nonsense_Mutation',

'Multi_Hit',

'Frame_Shift_Ins',

'In_Frame_Ins',

'Splice_Site',

'In_Frame_Del'

)

#查看变异类型对应的颜色

print(vc_cols)

oncostrip(maf = maf, top = 20)

maf.titv = titv(maf = maf, plot = FALSE, useSyn = TRUE)

plotTiTv(res = maf.titv)
